# Do histological variants in urothelial carcinoma of the bladder portend poor prognosis? A systematic review and meta-analysis

**DOI:** 10.18632/oncotarget.17593

**Published:** 2017-05-03

**Authors:** Qingke Chen, Lei Li, Gongxian Wang, Jieping Hu, Ting Sun, Bin Fu

**Affiliations:** ^1^ Department of Urology, The First Affiliated Hospital of Nanchang University, Nanchang, Jiangxi 330006, China; ^2^ Department of Anesthesiology, The First Affiliated Hospital of Nanchang University, Nanchang, Jiangxi 330006, China

**Keywords:** histological variants, squamous differentiation, urothelial carcinoma of the bladder, prognosis

## Abstract

The clinical implications of histological variants in urothelial carcinoma of the bladder has been a subject of significant controversy with many unanswered questions that remain. To clarify whether histological variants presage poor prognosis for patients suffering from urothelial carcinoma of the bladder, we scoured through various electronic databases such as Medline, Web of Knowledge, and the Cochrane Library up to August 18, 2016. Experts were consulted, and references from relevant articles were scanned. We identified thirteen eligible studies which met the inclusion criteria, including 9,533 participants. The existing evidence indicates that histological variants in urothelial carcinoma of the bladder patients do not alter their prognosis.

## INTRODUCTION

Bladder cancer has become a common cancer globally. An estimated 430, 000 new cases were diagnosed and 165, 000 deaths were recorded worldwide in 2012 [1]. Bladder cancer is clinically heterogeneous and characterized by non-muscle-invasive lesions that frequently recur, but are not associated with mortality. This is also in addition to aggressive muscle-invasive lesions associated with poor outcomes [2]. Endoscopic resection, chemotherapy or immunotherapy, photochemical internalization, and radical cystectomy are therapeutic strategies used to battle the disease [3–6].

Optimal treatment decisions must incorporate both clinical and pathological findings. Urothelial carcinoma has long been known to possess a remarkable propensity for divergent differentiation. Common morphologic manifestations of divergent differentiation appear along the squamous, glandular, small cell, and even trophoblastic lines. The incidence of divergent differentiation in cystectomy specimens is as high as 33%. Its presence is associated with established predictors of aggressive behavior [7]. Studies have suggested that the presence of histological variants (HVs) are associated with cancer-specific mortality in patients with urothelial carcinoma of the bladder when treated with radical cystectomy or intravesical immunotherapy [8, 9], however, some do not support this conclusion [10–12].

The small population of cases with histological variants in studies weakens the strength of evidence, it is possible that different types of differentiation have distinct outcomes. Furthermore, using cancer specific survival (CSS) or overall survival (OS) to evaluate the outcomes of histology variants produces different results. Therefore, we conducted a pooled analysis of currently available studies. In addition, subgroup analyses were carried out according to type of differentiation, outcome and treatment.

## RESULTS

### Study selection and characteristics

**Figure 1:**
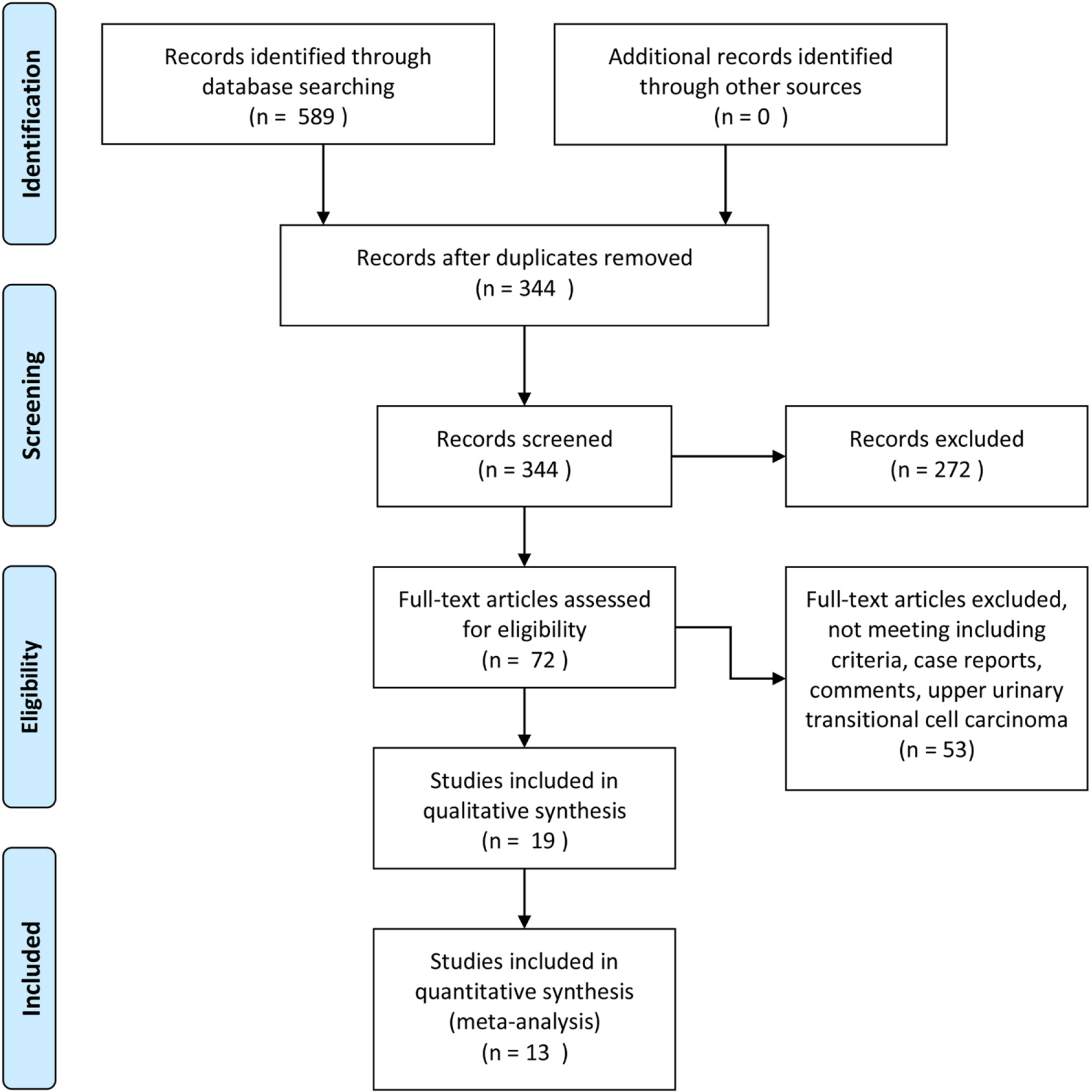
Flow diagram of search strategy to identify trials

A total of 589 potentially relevant papers were identified based on the search strategy. After removing duplicate records, 344 articles were screened by title and abstract, then 19 articles were retrieved in full-text for formal review. Thirteen studies with 1,823 cases and 7,710 controls were finally included in this meta-analysis [8–21]. The flow diagram of the literature retrieval and selection are shown in Figure 1. The characteristics of the studies included are listed in Table 1. A majority of patients in the studies underwent radical cystectomy, this is with the exception of one study where they received immunotherapy with BCG [9], and another study where they received mixed therapy including radiotherapy, chemotherapy or surgery [14]. Squamous differentiation (SD) was identified as the most common variant, however, many studies compared mixed histological variants (i.e. did not distinguish squamous, glandular, micropapillary, plasmacytoid, and sarcomatoid differentiation, apart from one study [17]) which looked at pure urothelial cancer of the bladder.

**Table 1:** Characteristics of the identified studies that met the predefined inclusion criteria

Study	Year	HV (cases)	PUCB (cases)	HR	LCI	UCI	Outcome	Treatment	HV (type)
Zargar-Shoshtari, K	2016	20	106	0.87	0.39	1.94	OS	RC	Mixed
Gofrit, O. N	2016	41	140	3.27	1.14	9.41	OS	BCG	Mixed
Soave, A	2015	96	389	1.46	1.07	1.99	CSS	RC	Mixed
Monn, M. F	2015	68	462	0.77	0.48	1.25	OS	RC	SD
Monn, M. F	2015	28	462	2.2	1.28	3.78	OS	RC	MPV
Monn, M. F	2015	25	462	2.42	1.33	4.42	OS	RC	PCV
Monn, M. F	2015	15	462	1.07	0.43	2.69	OS	RC	SAV
Monn, M. F	2015	26	462	0.92	0.44	1.91	OS	RC	Other
Izard, J. P	2015	325	2884	1.26	0.91	1.74	OS	RC	SD
Hsieh, M. C	2015	53	153	1.67	1.16	2.4	OS	Mixed	Mixed
Mitra, A. P	2014	141	141	0.92	0.77	1.09	OS	RC	SD
Mitra, A. P	2014	97	97	0.87	0.72	1.05	OS	RC	GD
Lee, Y. J	2014	51	645	1.52	1	2.32	OS	RC	Mixed
Xylinas, E	2013	261	1495	1.17	1.02	1.33	CSS	RC	Other
Xylinas, E	2013	227	1495	0.98	0.84	1.13	CSS	RC	SD
Kim, S. P	2012	186	827	0.79	0.6	1.05	CSS	RC	Mixed
Shapur, N. K	2011	79	144	2.37	1.18	4.78	OS	Mixed	Mixed
Antunes, A. A	2007	25	88	3.51	1.53	8.08	CSS	RC	SD
Scosyrev, E	2011	59	236	1.1	0.79	1.33	OS	RC	Mixed

### Risk of bias and grades of evidence

**Figure 2:**
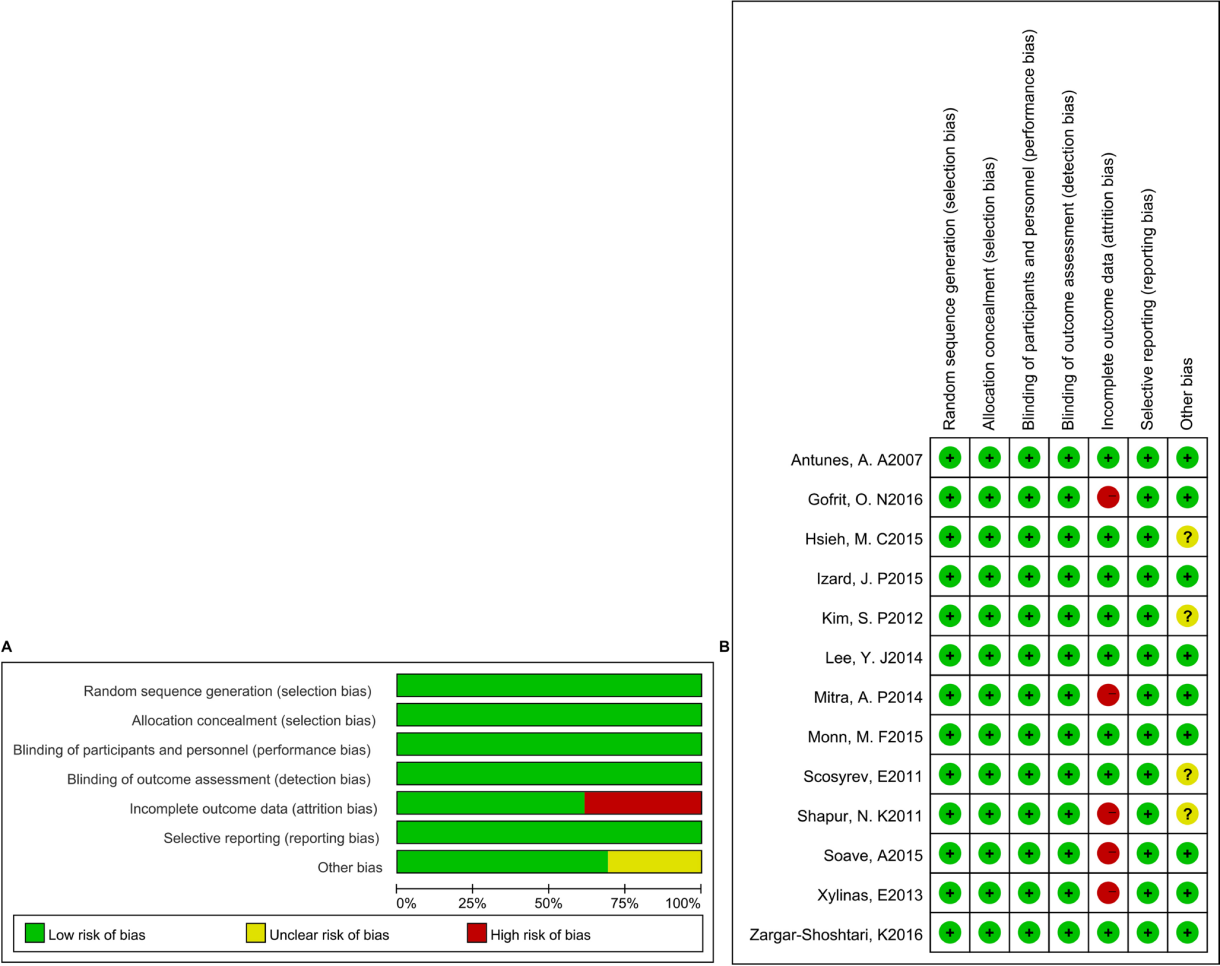
Risk of bias graph (**A**) and risk of bias summary (**B**).

The details for assessing risk of bias are shown in Figure 2. Thirteen trials were all open-label. Random sequence generation and allocation concealment were adequately performed in most of the trials. However, the data from five trials were extracted by Kaplan–Meier curve and read with Engauge Digitizer. It should be noted that the outcomes of the original trials may have a high risk of bias. The overall methodological quality of the included trials was generally good and fair. GRADE Working Group grades of evidence were high for OS and CSS.

### Data extractions and analysis

### Overall analysis

**Figure 3:**
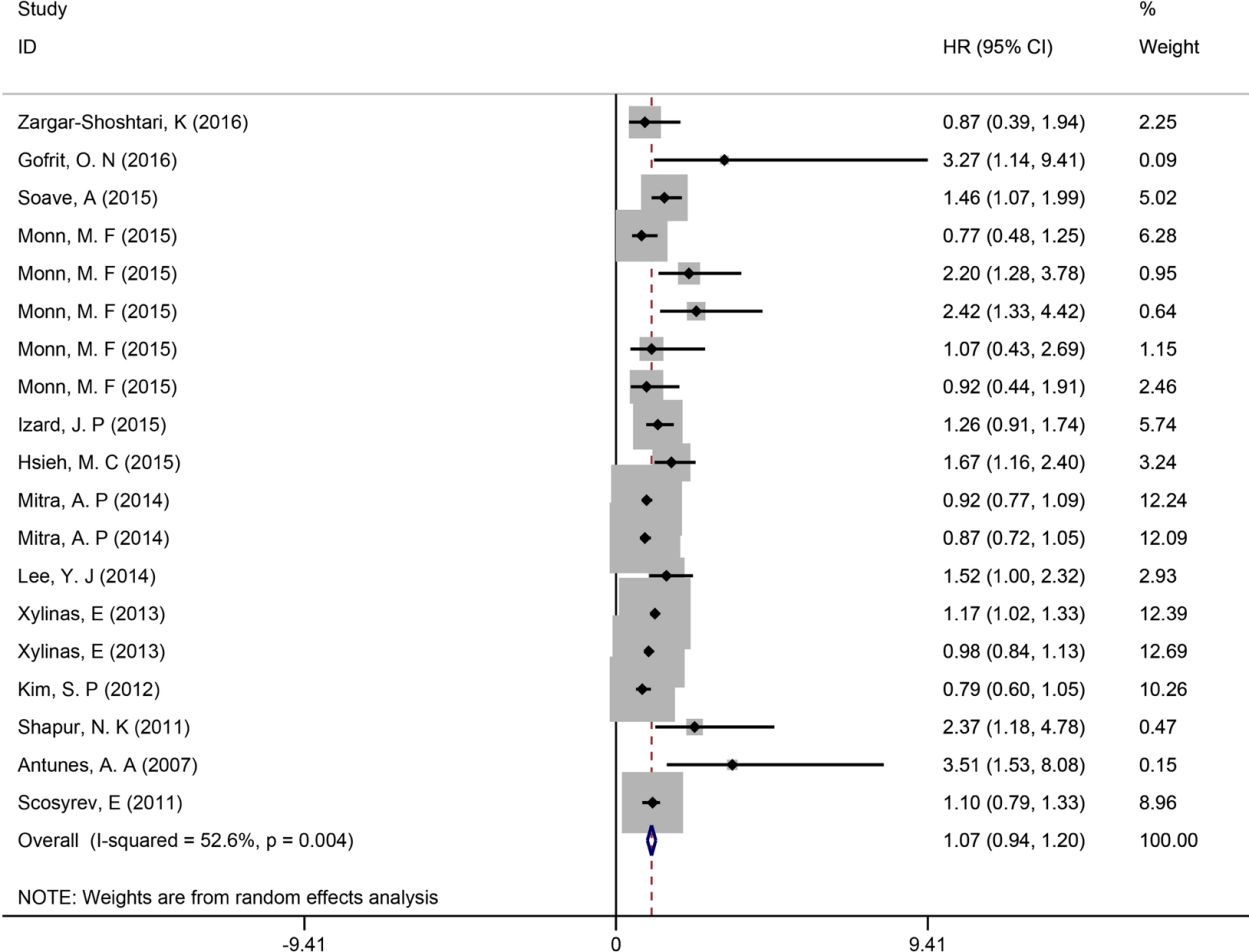
Overall analysis of HVs in UCB

Thirteen trials were analyzed, with a total of 9,533 participants who met inclusion criteria. HVs were observed in 1,823 of the 9,533 patients. Random effects model was applied as there was significant heterogeneity among these trials (*I*^2^ = 52.6%; *P* = 0.000). The pooled results showed that HV was not correlated with poor overall survival or cancer specific survival (HR = 1.07; 95% CI = 0.94, 1.20; *P* = 0.306; Figure 3).

### Subgroup analysis

**Figure 4:**
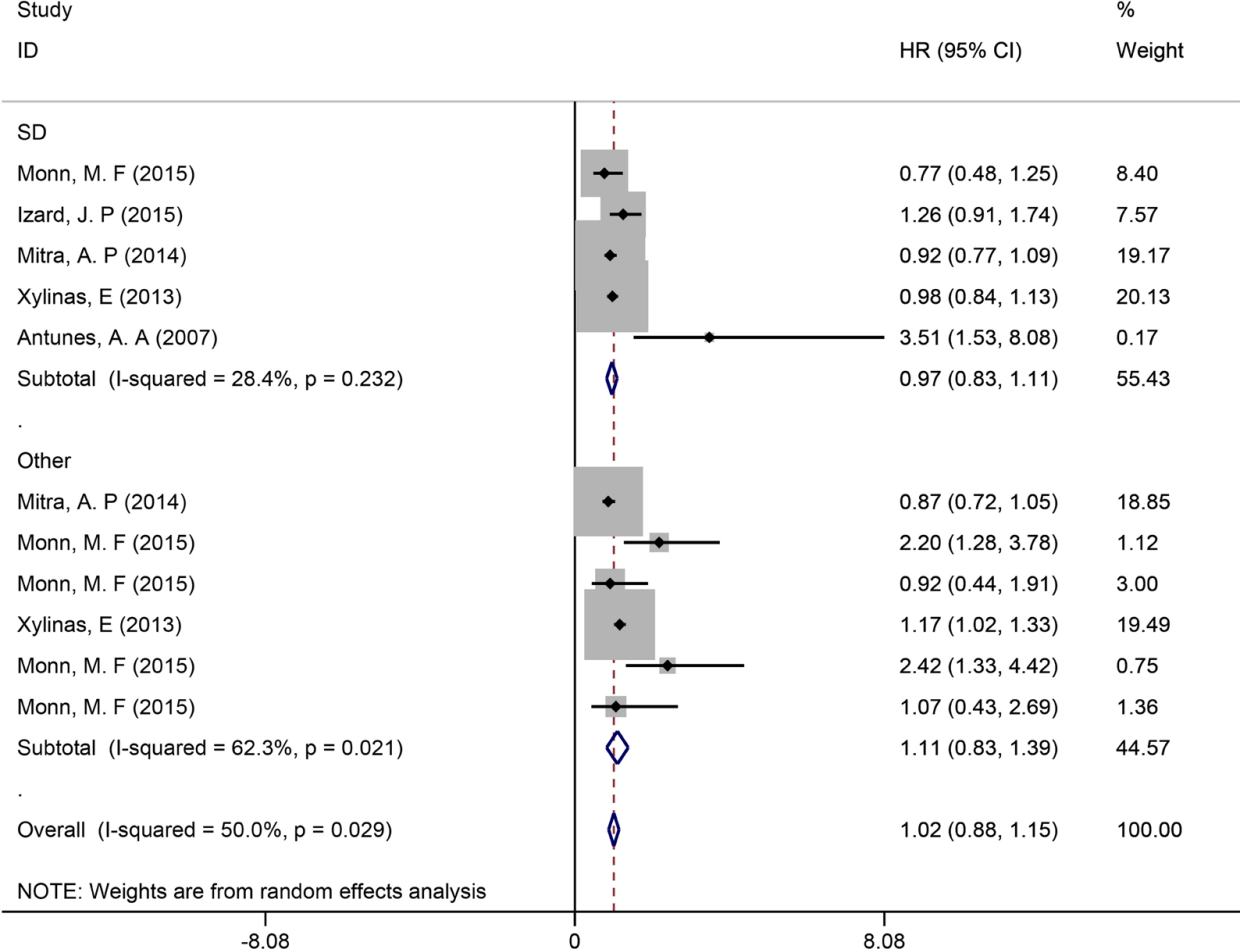
Subgroup analysis of survival in UCB patients with different HVs

Subgroup analysis was conducted according to the HV type, treatment, and endpoint (OS or CSS). Five studies with 786 SD cases (5,070 control cases) and 452 other HV cases (3440 control cases) were also analyzed. The meta-analysis indicated that the pooled HR was 0.97 (95% CI = 0.83, 1.11, *P* = 0.701; Figure 4) for SD which held no statistical significance. For other HVs, similar results were obtained (HR = 1.11, 95% CI = 0.83, 1.39, *P* = 0.481; Figure 4).

After the trials were stratified by treatment, ten studies with 1,670 HV cases and 10,621 PUCB cases treated by radical cystectomy (RC) underwent pooled analysis; the results suggested no significant difference between the two groups (HR = 1.03, 95% CI = 0.92, 1.15, *P* = 0.530; Supplementary Figure 1). Subgroup analysis for the two distinct endpoints were also stratified, nine studies with 1,028 HV cases and 6,856 PUCB cases were incorporated into an OS subgroup, while four studies with 795 HV cases and 4294 PUCB cases were incorporated into a CSS subgroup. The pooled results showed that there was no significant difference between the HV and PUCB cases regardless of OS (HR = 1.09, 95% CI = 0.92, 1.26, *P* = 0.319; Supplementary Figure 2) or CSS (HR = 1.06, 95% CI = 0.85, 1.28, *P* = 0.605; Supplementary Figure 2).

### Sensitivity analysis and publication bias

**Figure 5:**
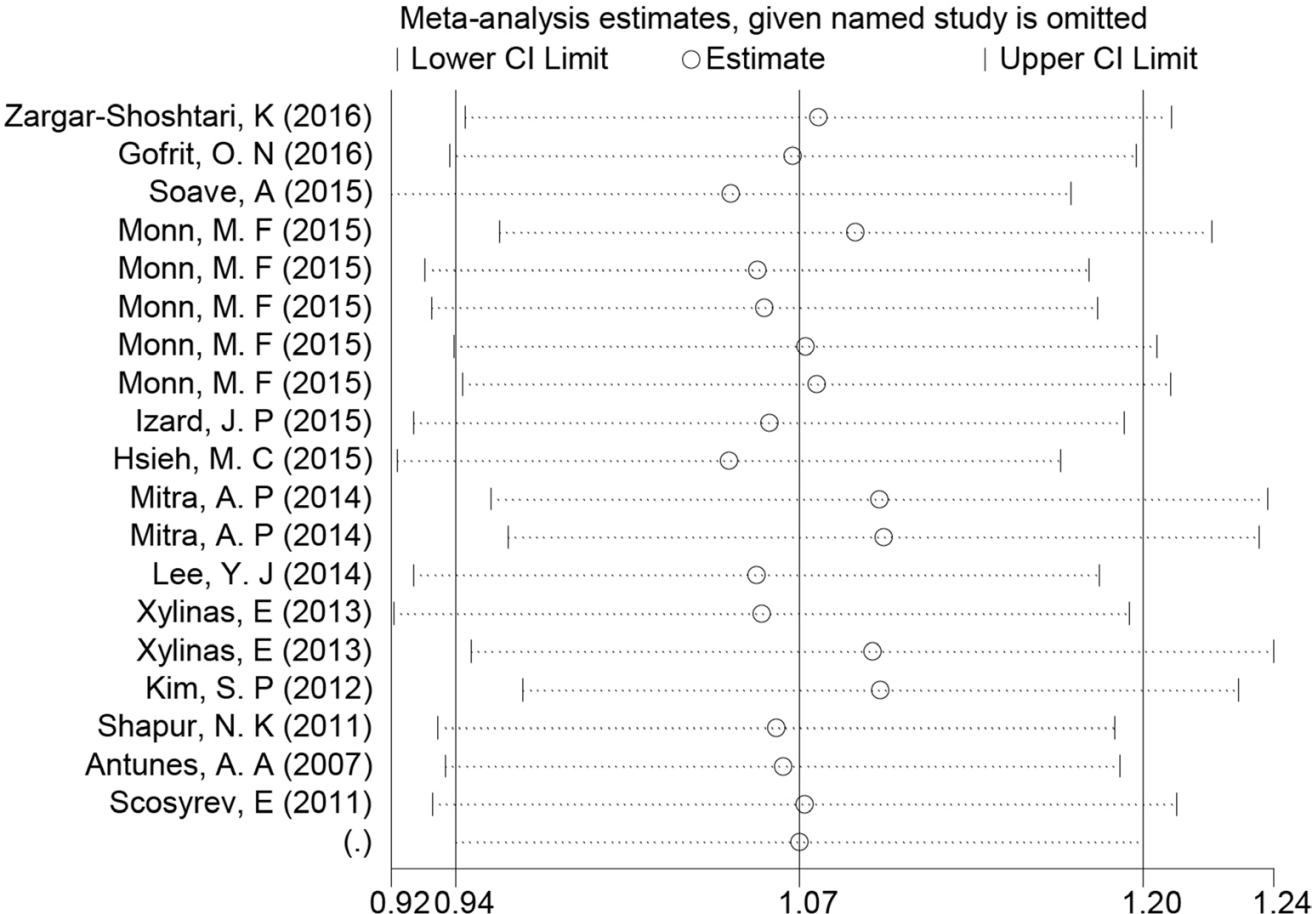
Sensitivity analysis of the summary HR of survival

One-way sensitivity analyses were performed to assess the stability of the pooled results. In the sensitivity analysis, each single study included in the meta-analysis was deleted each time to observe the influence of the data on the pooled ORs. No single study affected the pooled OR value, suggesting that the results of this meta-analysis were stable (Figure 5). Begger's funnel plot and Egger's test was performed to assess the publication bias [22]. The relatively symmetrical shape of the funnel plots provided evidence of a low level of publication bias (Supplementary Figure 3). Statistical analysis showed there was no significant publication bias for the Begger's test (Begger's test, *P* = 0.059; Egger's test, *P* = 0.022).

## DISCUSSION

In this systematic review and meta-analysis, we included thirteen trials with a total of 9,533 participants who suffered from UCB. Of these patients, 1,823 cases were pathologically confirmed UCB with HVs. GRADE Working Group grades of evidence ensure the high quality results of our analysis. The assessments of sensitivity and publication bias indicated that our analyses were reliable. Our results suggested that HV did not predict a poor prognosis. HVs should therefore be considered a negligible factor in a predictive model of survival.

Subgroup analysis indicated that squamous/ non-squamous differentiation did not influence survival. Antunes concluded that squamous differentiation was an independent prognostic factor for CSS in patients with bladder cancer treated by RC [13]. The small population of squamous differentiation (25 cases) rendered low weight (0.17%). Other studies have reported that squamous differentiation was associated with similar survival rates to PUCB [11, 12, 17, 20]. However, it should be noted that the presence of non-squamous differentiations such as micropapillary variant (MPV), plasmacytoid (PCV), and sarcomatoid (SAV) differentiation occurred in small populations of UCB patients, with studies suggesting that MPV and PCV act as the aggressive variants which are frequently associated with poor outcomes [23–25]. Therefore, the impact of these HVs on UCB patients’ survival should be evaluated with caution.

Radical cystectomy was the prevalent method of treatment for UCB patients with HVs. HVs did not influence the effects of RC; however, in patients who underwent mixed therapy [14, 18], HVs predicted poor prognosis (Supplementary Figure 1). In these studies, patients first underwent immunotherapy or chemotherapy and RC was offered to patients with disease progression, or to patients resistant to immunotherapy or chemotherapy. We expected patients with HVs to receive RC as early as possible to achieve a better outcome. Regarding OS or CSS, patients with HVs did not have different the outcomes, indicating current treatment strategies for patients with HVs are acceptable.

There are several limitations worth noting. Firstly, some studies have reached their conclusions from a small population [9, 10], so the quality of available research was perhaps compromised. Further studies consisting of a larger amount of patients are necessary to confirm these results. Secondly, the significance of HVs in various tumor stages were not evaluated. Available data was unable to analyse the meaning of various HVs in the same stage. Thirdly, the extent of variant histology was not assessed in most of the studied literature and the survival of mixed HV patients, (indicates the UCB accompanied by two or more variants), was not evaluated. Fourthly, radical cystectomy was the main treatment in most of the studies included, so the clinical significance of HVs in chemotherapy or immunotherapy require further exploration. Moreover, some variants have significant treatment implications—small cell needs neoadjuvant chemotherapy [26], micro papillary is fairly BCG resistant [9], etc. Overall, the majority of studies demonstrate that variant histology is more aggressive but combination treatments play a role in the final outcome. Furthermore, some cases have mixed variants versus pure.

## MATERIALS AND METHODS

### Systematic search strategy

We conducted a systematic search of electronic databases, including Medline, Web of Knowledge, and the Cochrane Library (updated August 18, 2016), to identify all relevant studies. The search combined key words: (‘‘Bladder cancer’’ OR ‘‘Urothelial carcinoma of the bladder’’) AND (‘‘Histological variants’’ OR ‘‘Squamous differentiation’’ OR ‘‘Glandular differentiation’’). See Appendix. Experts were consulted, and references from relevant articles were scanned. Studies were considered regardless of language status. Two independent authors (Qingke Chen and Jieping Hu) performed all aspects of the search strategy, screening the titles and abstracts of all articles following the inclusion criteria and then reviewing the full-text articles in detail.

### Identification of articles and data extractions

Trials performed with radical cystectomy, intravesical therapy or immunotherapy to compare histological variants (HV) and pure urothelial carcinoma of the bladder (PUCB) were eligible for inclusion. Two investigators independently extracted the data, and an agreement was reached through discussion. Eligible cases met the following criteria: (1) the diagnosis of urothelial carcinoma of the bladder had to be confirmed pathologically; (2) all patients should be confirmed as PUCB or UCB with HV; (3) patients underwent schedule immunotherapy, intravesical chemotherapy or radical cystectomy; (4) outcomes: overall survival (OS), objective response rate (ORR), progress-free survival (PFS) or cancer specific survival (CSS) were evaluated; and (5) study design was randomized controlled trial (RCT). The exclusion criteria for our analysis were as follows: (1) duplicated data (these were removed and only the updated data were selected); (2) patients with a history of previous urothelial carcinoma and concomitant upper tract urothelial carcinoma; (3) patients who underwent irregular intravesical chemotherapy or loss of follow up. The formula recommended by Spotswood et al. [27] was adopted to calculate the corresponding HR of the missing data (Supplementary Table 1). The Kaplan–Meier curve was read using Engauge Digitizer version 4.1 (available at: http://sourceforge.net/) unless the adequate data could be extracted [28].

### Assessing risk of bias and grading the quality of evidence

Assessment for the risk of bias was performed in accordance with the guidelines outlined in the Cochrane handbook for systematic reviews of interventions [29]. Two investigators (Qingke Chen and Jieping Hu) objectively reviewed all studies and assigned a value of “high”, “low” or “unclear” to the following domains: random sequence generation; allocation concealment; blinding of participants and personnel; blinding of outcome assessment; incomplete outcome data; selective reporting; and other biases. Trials with high risk of bias for any one or more key domains were considered to be “high risk”. Trial with low risk of bias for all key domains was considered to be “low risk”. Otherwise, they were considered as “unclear”[30].

The GRADE system identified four grades for rating the quality of evidence: high, moderate, low, and very low. The grade indicated the extent of further research required to change our attitudes concerning the estimate of the effect. GRADE profiler software (version 3.6) was utilized to rate the level of evidence.

### Data synthesis and data analysis

We estimated the HR or relative risk (RR) with 95% confidence interval (CI) for dichotomous outcomes, and the weighted mean difference (WMD) with 95% CI for continuous outcomes. Statistical heterogeneity among studies was evaluated utilizing *I*^2^ statistics (ranges from 0 to 100%), λ^2^ test, and *P* values[31]. The fixed effects model method (Mantel-Haenszel) was used, except in the case where a significant *Q* test (*P* < 0.05) or *I*^2^ > 50% indicated the existence of heterogeneity among studies. When the existence of heterogeneity was indicated, the random effects model (DerSimonian-Laird method) was instead applied [32]. The presence of publication bias was also evaluated using Begg and Egger tests. Sensitivity analysis was performed to assess the stability of the results. Funnel plots were drawn to estimate any potential publication bias, where the standard error of log (HR) of each study was plotted against its log (HR). Whether the funnel plot was symmetrical was assessed with the Egger's test [33, 34]. When using Egger's test to assess the publication bias, *P* < 0.05 was considered statistically significant. All statistical tests for this meta-analysis were performed with STATA 12.0.

## APPENDIX

(("urinary bladder neoplasms"[MeSH Terms] OR ("urinary"[All Fields] AND "bladder"[All Fields] AND "neoplasms"[All Fields]) OR "urinary bladder neoplasms"[All Fields] OR ("bladder"[All Fields] AND "cancer"[All Fields]) OR "bladder cancer"[All Fields]) OR (("carcinoma, transitional cell"[MeSH Terms] OR ("carcinoma"[All Fields] AND "transitional"[All Fields] AND "cell"[All Fields]) OR "transitional cell carcinoma"[All Fields] OR ("urothelial"[All Fields] AND "carcinoma"[All Fields]) OR "urothelial carcinoma"[All Fields]) AND ("urinary bladder"[MeSH Terms] OR ("urinary"[All Fields] AND "bladder"[All Fields]) OR "urinary bladder"[All Fields] OR "bladder"[All Fields]))) AND ((histological[All Fields] AND variants[All Fields]) OR (squamous[All Fields] AND ("cell differentiation"[MeSH Terms] OR ("cell"[All Fields] AND "differentiation"[All Fields]) OR "cell differentiation"[All Fields] OR "differentiation"[All Fields])) OR (glandular[All Fields] AND ("cell differentiation"[MeSH Terms] OR ("cell"[All Fields] AND "differentiation"[All Fields]) OR "cell differentiation"[All Fields] OR "differentiation"[All Fields]))).

## CONCLUSIONS

In summary, our study evaluated the roles of HVs in UCB patients. Our meta-analysis indicated that HVs did not predict poor prognosis for UCB patients who underwent RC. However, data showed that HVs were significantly associated with inferior survival for patients undergoing mixed therapy. Patients with HVs who receive RC as early as possible may achieve a better outcome.

## SUPPLEMENTARY MATERIALS FIGURES AND TABLES




