# Supplementary material for: Do histological variants in urothelial carcinoma of the bladder portend poor prognosis? A systematic review and meta-analysis
Source: Oncotarget. 2017 May 3;8(29):48263–71. doi: 10.18632/oncotarget.17593 (PMC5564644; doi:10.18632/oncotarget.17593)
Supplement: Supplementary file 1 [file oncotarget-08-48263-s001.pdf]

Do histological variants in urothelial carcinoma of the bladder portend poor prognosis? A systematic review and meta-analysis

Supplementary Materials

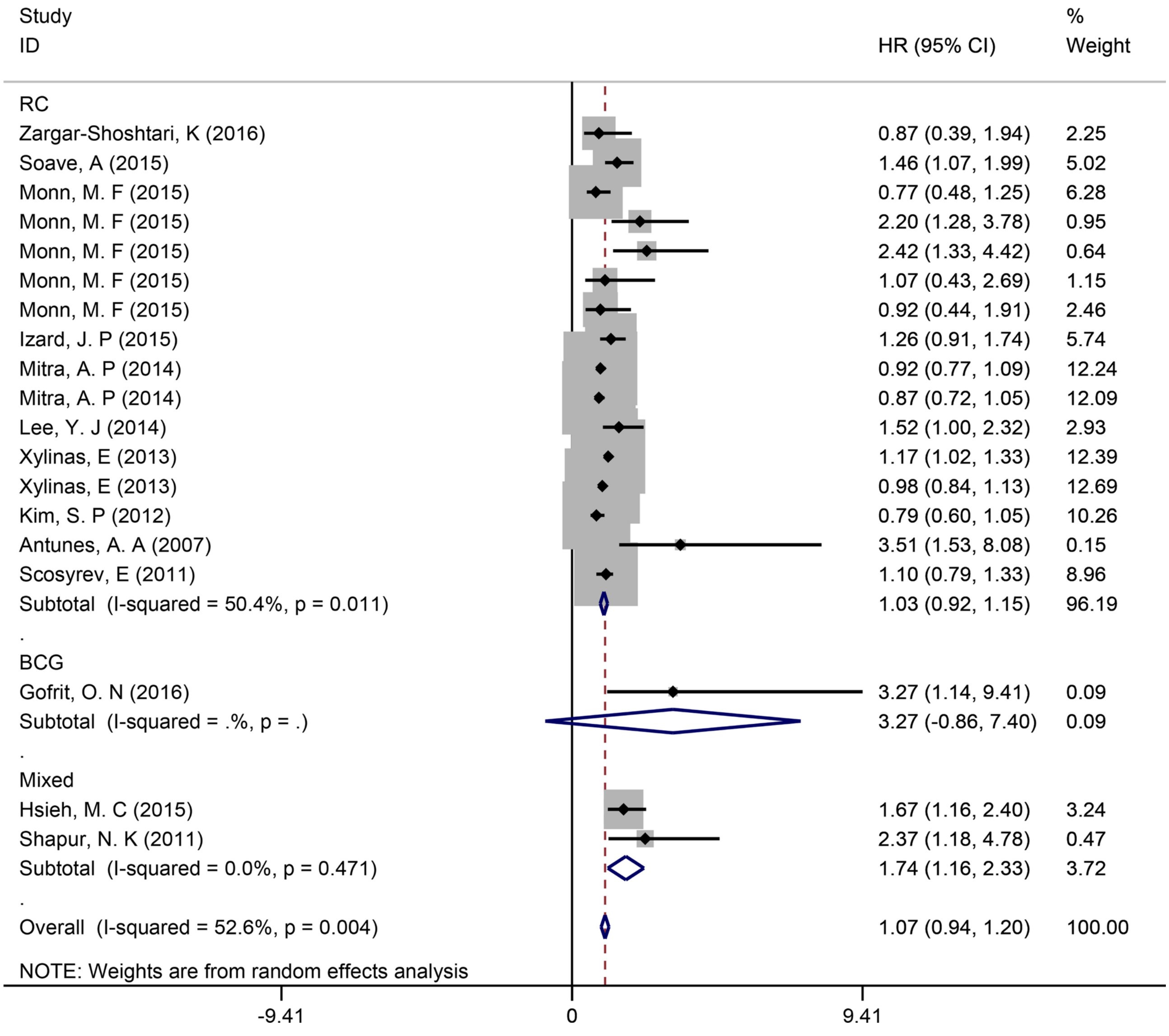

Supplementary Figure 1: Subgroup analysis of different treatments with HVs.

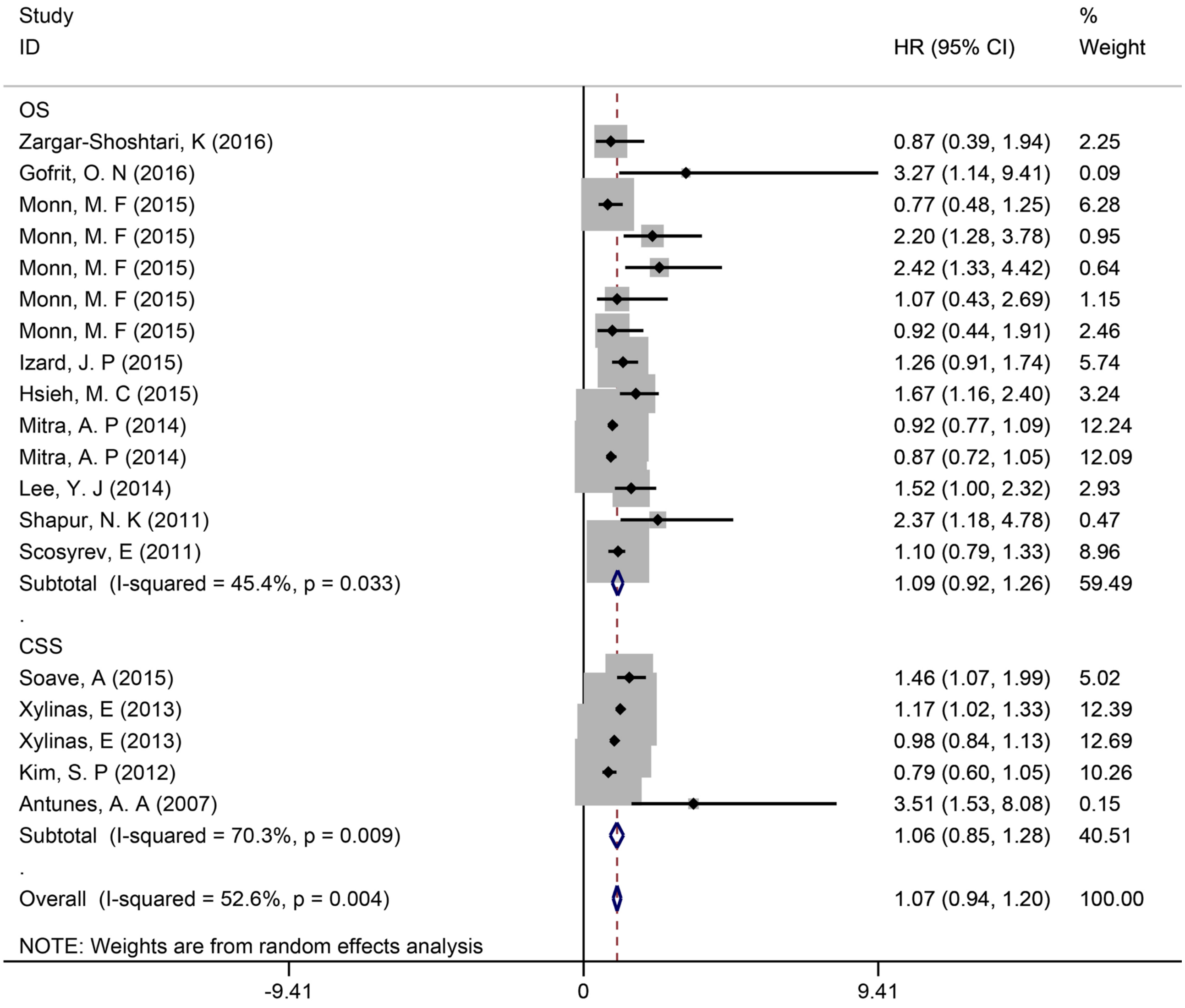

Supplementary Figure 2: Subgroup analysis of different endpoints with HVs.

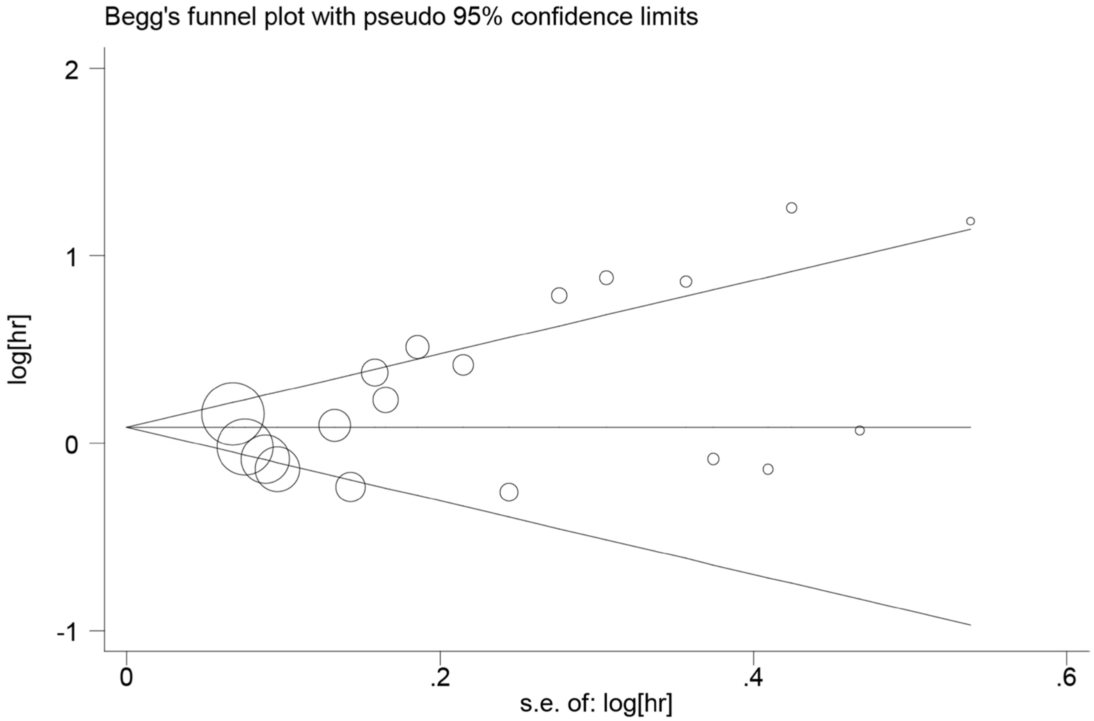

Supplementary Figure 3: Funnel plots of overall analysis.

Supplementary Table 1: The formula recommended by Spotswood et al. to calculate the corresponding HR. See Supplementary_Table_1
